# Agricultural production diversity, child dietary diversity and nutritional status in poor, rural Gansu Province of China

**DOI:** 10.1371/journal.pone.0287000

**Published:** 2023-06-14

**Authors:** Xinghua Liu, Chengfang Liu, Kevin Chen

**Affiliations:** 1 China Academy for Rural Development, Zhejiang University, Hangzhou, China; 2 China Center for Agricultural Policy, School of Advanced Agricultural Sciences, Peking University, Beijing, China; 3 International Food Policy Research Institute, East and Central Asia Office, Beijing, China; University of Kara, TOGO

## Abstract

Research has shown mixed findings on the link between production diversity and household dietary diversity. The question is whether this link holds for children. In this study we examine the relationship between household’s agricultural production diversity and child dietary diversity, and between production diversity and child nutritional status. Smallholder farm households (n = 1067) and children (n = 1067) aged 3–16 years from two then nationally designated poverty counties in Gansu Province of China were interviewed in 2019. Production diversity was assessed with the production richness score and production diversity score. Production diversity was calculated from agricultural production data covering a period of 12 months. Child dietary diversity was assessed with food variety score (FVS) and dietary diversity score (DDS). DDS was calculated based on 9 food groups using a 30-day recall method. Data were analysed using Poisson and Probit regression models. We find that both agricultural production richness score and revenue generated from selling agricultural products are positively associated with food variety score, with the relationship being stronger for the latter. Moreover, production diversity score is positively associated with children’s dietary diversity score whereas negatively associated with their probability of being stunted, but not with their probabilities of being wasted or zinc deficient. Household social economics status were also positively associated with child dietary diversity.

## Introduction

It was estimated that 200 million children aged less than five years were stunted or wasted and 340 million children suffered from deficiency of essential vitamins and minerals globally in 2019 [1]. Child undernutrition has lifelong health, social and economic consequences, negatively affecting health status, early childhood cognitive development, learning outcomes in school, and individual productivity and earning potential in adulthood [2, 3]. Previous studies have suggested a positive impact of dietary diversity on children’s nutritional status [4, 5]. While there is a heated debate around whether market-oriented agricultural production specialization or farm diversification plays a larger role in enhancing dietary diversity, the latter is often considered as a more direct route, especially for smallholder subsistence farm households in less developed areas [6, 7].

An emerging body of literature has examined the link between production diversity and dietary diversity and the results are mixed and context-specific [8]. A small number of studies has shown farm production diversity is consistently positively and significantly linked to dietary diversity at household or individual level [6]. Diversified agricultural production contributes to dietary diversity and nutrition security mainly through two pathways [9]. One is subsistence pathway where diversified agricultural production directly contributes to food and nutrition security and diverse diets. The other is income-generating pathway where revenues generated from selling agricultural products in markets provide farmers with income to buy nutritious food and increase their diet diversity. The majority of existing studies, however, find that significant relationship only holds for certain subsamples or using certain measures of production diversity and/or dietary diversity [10]. Moreover, the magnitude of the positive association is relatively small with variations by regions. A meta-analysis indicates that 16 additional species have to be produced by farm households in order to increase their diet by one more food group [7]. And the association varies by regions, with larger mean effect in Sub-Saharan Africa than other regions. Nonetheless, African farmers would still have to produce around 9 additional species to increase dietary diversity by one food group. In contrast, some studies found null relationship regardless of subsamples or indicators used [11]. Still other studies even found that when diversity exceeds a certain threshold, further additions of the species can lead to income and dietary diversity reduction as a result of loss of economies of scale [12, 13].

This study aims to examine the associations between household production diversity and children’s dietary diversity as well as their nutritional status, using data from two then nationally designated poverty counties in Gansu province of China. Specifically, we sought to answer the following two research questions. First, is household production diversity associated with child dietary diversity? Second, is household production diversity associated with improved child nutritional status?

This study contributes to the literature in two ways. First, in this study, we focus specifically on child dietary diversity and link it with child nutritional status. This offers additional insight into how farm diversity is associated with dietary quality and nutritional status of children in the farm households. Although there have been studies on the link between production diversity and dietary diversity at household level, few studies have focused on the dietary quality of a particular vulnerable group of household members, such as children. Second, in addition to the specific link between farm diversity and children’s dietary diversity among rural households in less developed areas in China, this study also examines the role that market orientation and off-farm employment play in enhancing child dietary diversity and nutritional status. This provides new evidence on the relationship between farm diversity and child dietary diversity in the context of poor rural China.

## Materials and methods

### Samples

This study draws on data collected by the authors themselves in September 2019 as part of a baseline survey under the project of Gansu Smallholder Farmers Growing Zinc-enriched Potatoes Pilot commissioned by the World Food Programme (WFP). One key objective of the pilot is to increase the availability of zinc-rich potatoes in poor, rural Gansu. The baseline survey aims to collect rich and detailed information on the health and nutritional status of children in selected districts in an attempt to better inform intervention design to reduce potential zinc deficiency in the study area. The baseline survey was conducted prior to any intervention.

The WFP project office pre-selected 4 township primary schools and 4 preschools in Gansu Province of China. One is Anding District in Dingxi City and the other Dongxiang County in Linxia Hui Autonomous Prefecture. The survey team surveyed primary students aged 6–13 years (n = 868) and preschoolers aged 3–6 years (n = 412) who were present in schools on the survey date in a one-on-one, face-to-face manner. In total, we surveyed 1,280 caregivers. Accordingly, we should have surveyed 1,280 children. As it turned out, we collected data on height, weight and hair sample for 1,178 children (or 92%). The rest 102 children (or 8%) did not attend the module for anthropometric measures. After further excluding observations with missing data on certain control variables, we have 1,067 children in our final sample for further analysis.

### Child dietary diversity measurement

Child dietary data were obtained by trained enumerators through asking the primary caregivers (mostly grandparents or parents) what the child ate at home as well as other places (e.g., schools, restaurants, shops) over the past 30 days. Unlike previous studies using 24-hour recall period [14, 15], this study draws on data on the range of foods eaten over a longer reference period (i.e. the 30-day period). This enables us to capture more of the day-to-day variation in individual food consumption [10]. That said, the understate of the overall dietary diversity is less of a concern for this study.

Our dietary assessment questionnaire was based on the FAO guidelines for measuring household and individual dietary diversity [16]. The questionnaire included 75 food items/food groups which were then aggregated into the nine food group as proposed by FAO. The nine food groups are: cereals; white tubers and roots; legumes, legume products, nuts, and seeds; vegetables and vegetable products; fruits; meat; eggs; fish and fish products; and milk and milk products.

Child dietary diversity was computed using both food variety score (FVS) and child dietary diversity score (DDS). FVS counts the number of food items consumed by the child over a 30-day period. DDS, however, counts the number of food groups; thus food items belonging to the same food group will only be counted once.

### Child nutritional status measurement

Children’s nutritional status was measured by anthropometric indicators. Children’s height and weight was measured by trained nurses on site in accordance with the WHO standard protocol [17]. The nursing team was trained to ensure that the weighing station was set up on level ground to ensure accuracy of the equipment. The children were measured in light clothing without shoes, hats, or accessories. Height was recorded to the nearest 0.1 cm with standard tape measure, and weight was recorded to the nearest 0.1 kg using a calibrated electronic scale. Zinc status was analysed using hair zinc. Hair samples were collected on site with stainless steel scissors from the occipitonasal region of the child’s head, within 3 cm of the hair line. These samples were stored in chemical-free, polypropylene containers and were later sent to the lab in the Civil Aviation General Hospital for an analysis of the zinc level.

Height and weight were used to construct height-for-age z-scores (HAZ), weight-for-height z-scores (WHZ), and (BMI)-for-age z-score (BmiAZ), using new 2006 WHO child growth standards. Following internationally recognised cut-offs [18], children were considered stunted or wasted if his/her HAZ or WHZ (applies for children under 61 months)/BmiAZ (applies for children over 61 months) to fall more than two standard deviations below the the WHO Child Growth Standards median, respectively.

Zinc status is another nutritional indicator of interest given the high zinc deficiency prevalence in our sample area and the aforementioned ***pilot*** objective. Zinc status was determined based on hair zinc analysis. Note that concentrations of zinc in hair are considered to be more stable and can reflect zinc level in a longer period of time compared with serum zinc [19]. Following the definition by the Trace Elements Science Association of China for zinc deficiency, zinc deficiency was defined as hair zinc level smaller than 90 μg/g for children aged between 0 and 18 years. See Table 1 for the definition and formulas of the nutritional indicators.

**Table 1:** Descriptive statistics of key variables.

	Definition	Unit	Mean or %	SD
***Dependent variable***				
*Child dietary diversity*				
Food variety score	Number of food items consumed over past 30 days	Number	22.64	9.78
Dietary diversity score	Number of food groups consumed over past 30 days	Number	6.60	1.31
*Child nutritional status*				
Stunting	Height-for-age Z-scores<-2 SD	(0,1)	10.87	-
Wasting	Weight-for-height Z-scores<-2 SD	(0,1)	7.90	-
Zinc deficient	Hair zinc level < 90 μg/g	(0,1)	20.73	-
***Independent variable***				
*Production diversity*				
Production richness score	Number of crop and livestock species farm produced	Number	3.71	2.14
Production diversity score	Number of different food groups produced on a farm	Number	3.29	1.56
***Control variables***				
*Child level*				
Age	Age in years	years	7.84	2.78
Gender	1 = Boy; 0 = Girl	(0,1)	50.33	-
Ethnicity	1 = Han; 0 = Other	(0,1)	56.89	-
Siblings	Number of siblings	Number	1.83	1.22
Left-behind status	1 = Left-behind; 0 = Non left-behind	(0,1)	66.82	-
*Household level*				
Household size	Household size	Number	5.15	1.50
Land cultivated	Cultivated land area	Mu	6.74	6.99
Market participation	Share of crop produce sold to market	%	0.13	0.21
Remittance (log)	Money sent back home from migrant workers	yuan/year	3.89	6.89
Consumption durables	Items of household durables owned	Number	3.08	1.43
Business ownership	1 = Own business; 0 = Otherwise	(0,1)	6.84	-
Number of housing	Number of housing owned	Number	1.14	0.49
Toilet	1 = Flush toilet; 0 = Otherwise	(0,1)	10.12	-
Drinking water	1 = Tap water; 0 = Otherwise	(0,1)	86.50	-
Kitchen	1 = Separate kitchen; 0 = Otherwise	(0,1)	78.73	-
*Primary caregiver level*				
Age	Age in years	years	38.11	12.03
Educational attainment	Years of education	years	2.88	3.82

### Production diversity measurement

Household agricultural production diversity data were obtained by asking caregivers the varieties of crops planted and livestock raised by the household over the past 12 months. Production diversity was measured by production richness score and production diversity score [20]. Production richness score is computed by simply counting the overall number of species, including both crop and livestock, that were farm produced. Production diversity score is calculated based on the number of different food groups produced on a farm. We use the same 9 food groups as aforementioned for DDS on the consumption side.

### Control variables

Following previous literature, child-, household- and primary caregiver- level characteristics were included as control variables.

Previous literature suggests that circumstance factors that are beyond the control of individual child could affect child undernutrition and dietary quality [21]. As such, child-level characteristics including child’s age, gender, ethnicity, number of sibling and left behind status were added in our model to control for such circumstance factors.

In addition, existing literature has shown that the number of household members, market oriented agricultural production and off-farm income are positively associated with dietary diversity and nutritional status [21, 22]. To account for such factors, we control for household-level characteristics including household size, cultivated land area, the share of crop produce sold to market and remittance from migrant workers.

Motived by a substantial body of empirical studies examining the association between household socioeconomic status, parental background and children’s development [15, 21, 23, 24], we included in our empirical model household-level characteristics (i.e. number of household consumption durable items, business ownership, number of housing, whether household has tap water, flush toilet and a separate kitchen), as well as primary caregiver-level characteristics (i.e. primary caregiver’s age, educational attainment).

### Statistical analysis

Descriptive statistics were used to describe household production diversity, child dietary diversity, child anthropometric variables and covariates. We presented mean and SD for continuous variables whereas frequencies for binary/categorical variables.

Following the literature [22], we use Poisson model to examine the relationship between production diversity and children’s dietary diversity as dietary diversity is measured by a count variable, which follows a Poisson distribution. Linear regression will not be appropriate in this case as it assumes normal distribution. The Poisson model is specified as follows,

$${DD}_{i}=\alpha_{0}+\alpha_{1}{PD}_{i}+\alpha_{2}{{PD}_{i}}^{2}+X_{i}+\varepsilon_{i}$$ (1)

Where *DD*_*i*_ denotes the dietary diversity of child *i*, which is measured by food variety score (FVS) or child dietary diversity score (DDS). *PD*_*i*_ is the production diversity of household *i*. *PD*_*i*_^2^, the square team of production diversity, is included to test whether there exists any inverted U-shape relationship between household production diversity and children’s dietary diversity, which has been reported in prior studies [12]. *X*_*i*_ is a vector of control variables as mentioned above. *ε*_*i*_ is the error term.

We use Probit model to examine the relationship between production diversity and children’s nutritional status. The reason for using Probit model is that we follow the literature and measure child nutrition status by a set of binary variables, including stunting, wasting and zinc deficiency [20]. When the left-hand side variable is binary, it is common to use Probit model as the linear probability model based on OLS might produce predictions less than zero or greater than one. The Probit model is specified as follows:

$$N_{i}=\alpha_{0}+\alpha_{1}{PD}_{i}{+X}_{i}+\varepsilon_{i}$$ (2)

Where *N*_*i*_ denotes the nutritional status of child *i*, which is measured by three dummy variables indicating whether a child is stunted, wasted or zinc deficient, respectively. Other notations are the same as in Eq (1). *α*_1_ captures the coefficient of interest. All data analyses were conducted in STATA Version 17. P<0.05 was considered statistically significant.

### Ethics statement

The standard survey protocols, questionnaires and procedures of anthropometry was approved by the Institutional Review Board (IRB) at IFPRI Headquarter and at Peking University Health Science Centre. All the caregivers of sample children were informed that participation was voluntary and were assured about confidentiality of the information they would provide during the interview. Prior to the interview, informed consent was obtained from each participant. Trained enumerators ensured that the caregivers were fully aware of related risks, especially those in the physical examination. The study values the privacy, confidentiality and anonymity of participants. Although during the field survey enumerators wrote down the participants’ names on questionnaires notes, those are for internal use only. No data, view, or statement will be attributed to a named individual, or presented to external audiences in such a way that an individual can be traced as its source.

## Results

A total of 1,280 caregivers were interviewed with caregiver questionnaires. Anthropometric measurements, i.e. height and weight were done for 1,178 children. After excluding those with missing entries of key variables, the final sample is 1,067. Note that sample sizes in the tables in what follows are different for different outcome variable depending on data availability.

Table 1 presents the descriptive statistics on key variables. On average, children consumed 22.64 species in the 30-day proceeding the interview. Children consumed an average of 6.60 food groups during sample period. For the binary indicators of child nutritional status, 10.87% were stunted, 7.9% were wasted and 20.73% were suffering from zinc deficiency. On the production side, households produced an average of 3.71 species, both crop and animal species included, as indicated by the production richness score. The production diversity score was 3.29, implying that a household produced an average of 3.29 different food groups in the past 12 months prior to the month of the interview.

Table 1 also shows that children’s average age was 7.84, half were male (50.33%), and 43.11% were ethnic minority. The children had an average of 1.83 siblings. Around two-thirds of them were left-behind children (66.82%). The mean household size was 5.15 (SD 1.5) and households cultivated an average of 6.74 mu land. The value of market-oriented production accounted for only about 13% of total production, which indicates that households in our sample predominantly practiced subsistence farming, that is farming for own consumption. The mean logarithmic term of remittance is 3.89. The households owned an average of 3.08 items of household consumption durables and 6.84% of them were business owners. They possessed an average of 1.14 number of housing and the proportion of households had access to flushable toilet, tap drinking water and private kitchen were 10.12%, 86.5% and 78.73%, respectively. The mean age of children’s primary caregiver was 38.11 years, and the education attainment was 2.88 years.

There were positive, statistically significant associations between production richness score and food variety score (P< 0.003) in the adjusted models (Table 2). Production diversity score was also positively associated with dietary diversity score (P <0.010) in the multivariate regression models. Some control variables show consistent associations with child dietary diversity. For example, the correlation between number of consumption durables and FVS or DDS was positive and significant. Similarly, having a private kitchen in the household was also linked to higher FVS or DDS. Conversely, land size was negatively associated with FVS and DDS and was statistically significant. Market participation, measured by the share of the value of agricultural production sold to market, was statistically significantly associated with FVS and its coefficient was larger than that of production richness score.

**Table 2:** Associations between household production diversity and child dietary diversity.

	Food variety score		Dietary diversity score	
	estimate	p value	estimate	p value
Production richness/diversity score	**2.25**	0.003	**0.51**	0.010
Square term of production diversity	-0.14	0.076	-0.05	0.079
Age (years)	-0.56	0.247	0.01	0.893
Male	0.04	0.658	0.01	0.759
Han	**3.70**	0.011	0.38	0.409
Number of siblings	-0.17	0.730	0.03	0.686
Left-behind children	0.41	0.833	-0.34	0.120
Household size	-0.16	0.492	-0.02	0.708
Land cultivated	**-0.17**	0.004	**-0.02**	0.048
Market participation	**4.43**	0.000	0.34	0.137
Remittance (yuan/year) (log)	-0.10	0.226	0.02	0.070
Number of consumption durables	**1.99**	0.000	**0.20**	0.000
Own business	**3.41**	0.025	0.18	0.445
Number of housing	0.79	0.356	0.04	0.572
Flush toilet	0.70	0.378	0.03	0.815
Tap water	-0.56	0.223	-0.20	0.123
Own separate kitchen	**1.79**	0.015	**0.37**	0.003
Primary caregiver’s age	0.00	0.820	0.00	0.776
Primary caregiver’s education	-0.14	0.246	0.00	0.958

Notes: Significance at the α < 0·05 level indicated using boldface type.

After adjustment, production diversity score was negatively associated with stunting (P < 0.008), but not wasting and zinc deficiency. However, production richness score was not associated with any of the three child nutritional status indicators (Table 3).

**Table 3:** Associations between household production diversity and child nutritional status.

	Stunting		Wasting		Zinc deficiency	
	estimate	p value	estimate	p value	estimate	p value
**Production richness score**	-0.01	0.252	-0.01	0.215	-0.01	0.197
Age (years)	0.00	0.900	0.00	0.999	**-0.05**	0.000
Male	**0.02**	0.020	**0.01**	0.017	-0.03	0.096
Han	-0.17	0.264	**0.51**	0.000	-0.20	0.245
Number of siblings	0.00	0.978	-0.01	0.308	0.01	0.088
Left-behind children	-0.03	0.62	-0.07	0.230	**0.15**	0.009
Household size	**0.02**	0.026	**0.02**	0.000	0.00	0.912
Land cultivated	**0.01**	0.002	**0.00**	0.013	0.00	0.530
Market participation	-0.09	0.169	-0.03	0.714	-0.09	0.271
Remittance (yuan/year) (log)	0.00	0.649	0.00	0.354	**-0.01**	0.005
Number of consumption durables	-0.01	0.133	**0.02**	0.011	0.02	0.248
Own business	-	-	0.03	0.682	0.11	0.431
Number of housing	**-0.05**	0.010	-0.04	0.072	0.02	0.577
Flush toilet	**-0.12**	0.046	-0.07	0.329	-0.03	0.720
Tap water	-0.03	0.505	**-0.09**	0.000	-0.05	0.081
Separate kitchen	-0.04	0.346	0.00	0.921	0.02	0.384
Primary caregiver’s age	0.00	0.182	0.00	0.918	0.00	0.104
Primary caregiver’s education	0.00	0.949	0.00	0.396	0.00	0.602
**Production diversity score**	**-0.03**	0.008	0.01	0.608	0.01	0.296
Age (years)	0.01	0.847	0.00	0.975	**-0.05**	0.001
Male	**0.02**	0.019	**0.01**	0.019	-0.03	0.093
Han	-0.18	0.250	**0.54**	0.000	-0.18	0.295
Number of siblings	0.00	0.997	-0.01	0.314	0.01	0.071
Left-behind children	-0.02	0.685	-0.07	0.190	**0.13**	0.031
Household size	**0.02**	0.022	**0.02**	0.000	0.00	0.895
Land cultivated	**0.01**	0.009	0.00	0.443	0.00	0.192
Market participation	-0.10	0.098	-0.01	0.910	-0.05	0.540
Remittance (yuan/year) (log)	0.00	0.638	0.00	0.298	**-0.01**	0.020
Number of consumption durables	-0.01	0.300	**0.02**	0.022	0.02	0.294
Own business	-	-	0.04	0.630	0.13	0.409
Number of housing	**-0.05**	0.010	-0.04	0.073	0.02	0.543
Flush toilet	-0.12	0.075	-0.08	0.270	-0.03	0.691
Tap water	-0.03	0.416	**-0.09**	0.000	-0.05	0.112
Separate kitchen	-0.04	0.251	0.00	0.887	0.02	0.284
Primary caregiver’s age	0.00	0.160	0.00	0.795	0.00	0.094
Primary caregiver’s education	0.00	0.963	0.00	0.401	-0.01	0.540

Notes: Significance at the α < 0·05 level indicated using boldface type. Note that hair samples were only available for 476 children.

## Discussions

A diversified diet is central to the nutrition of the majority of the poorest children in the world [25]. Intuitively and suggested by prior literature, agriculture can affect household nutritional status by producing food for own consumption and by generating agricultural revenues through market sales. To that end, promoting agricultural diversity is considered to be a potential pathway for achieving both nutrition security and agricultural biodiversity [26].

While the international evidence on the overall effects of agricultural production diversity on dietary diversity has been somewhat mixed, there is little evidence on its impacts on children. This paper contributes to the understanding of the relationship between production diversity and dietary diversity for children living in then impoverished rural area in China. We examined not only the association between production diversity and child dietary diversity, but also the link between production diversity and child nutritional status. Overall, our empirical analyses suggest that household farm production diversity was positively associated with dietary diversity in children. This finding is in agreement with prior studies which found that increased agricultural diversity, under certain cases, is associated with greater household dietary diversity [22, 27–30], yet adds to the current literature by demonstrating this cross-sectional association also holds true at child level. This finding also implies that promoting agricultural diversity should not be used as a stand-alone policy.

This study also highlights the role of markets in improving child dietary quality. This finding is consistent with empirical and theoretical work suggesting a higher dietary diversity level in market-oriented than in subsistence-oriented settings [22, 30]. Generally, greater market access or market sales revenue has been found to be linked to enhanced dietary diversity, and the effects are stronger than agricultural production diversity per se [10]. Our results align with theirs in that both agricultural production richness score and revenue generated from selling to market are positively associated with food variety score, while the relationship is stronger for the latter. Moreover, we found that revenue generated from agricultural market sale has a stronger relationship with child dietary diversity than production diversity. This is in agreement with prior studies demonstrating the importance of market access and market participation in enhancing food and nutrition security [9, 31, 32]. Indeed, the inverted U-shaped relationship between production diversity and child dietary diversity suggests that highly diversified systems may lead to lower income as a result of the loss in economies of scale and therefore leading to a lower dietary diversity [6, 10].

We also found that production diversity was negatively associated with stunting. This is in agreement with a cross-sectional study in Zambia, which found significant positive associations between production diversity and stunting among children aged 24–59 months [15]. However, our findings contradicted with previous studies which demonstrates higher ratios of own-consumption are associated with increased probabilities of stunting [23]. Similarly, it is also in contrast with evidence from rural Kenya which shows agricultural diversity is not linked with child stunted growth [33]. Overall, our findings signify that household production diversity plays a significant role in child nutrition, especially stunting.

In addition, our results show production diversity was not associated with wasting or zinc deficiency, adding to the current literature from the dimension of hidden hunger (micronutrient deficiency: zinc in this case). The lack of association between nutritional status indicators and the production diversity can be caused by the multifaceted nature of child malnutrition; thus suggesting a potentially more complex relationship between the two [34]. In addition, the lack of relationship with zinc deficiency may be because many of the food produced on the farm are low in zinc. This, to some extent, indicates the importance of producing nutrition fortification products such as zinc-enriched potatoes which will be the main intervention of the pilot used in this study.

As has been shown in the literature, childhood undernutrition is caused by a complex of factors. Consequently, health policies aimed at improving children’s dietary quality and nutritional outcomes should take a holistic approach and focus on those other factors as well. To that end, our findings support the evidence that other factors, such as having household consumption durables or having a separate kitchen is also important. This concurs with a prior study demonstrating that the number of assets owned by a household is significantly associated with increased HAZ scores [33]. This finding is also consistent with previous studies which document that factors other than production diversity also play a role in contributing to a balanced diet [35, 36] and may also affect the association between production diversity and dietary diversity. These factors include nutrition knowledge, education, gender, off-farm income, market access, use of certain farm and storage technologies [15, 20, 24].

We acknowledge at least three limitations of this study. First, constrained by data, this study relies on cross-sectional data to identify correlation. Future studies could draw upon panel data to identify causal relationship. Second, while seasonality is important, we were unable to account for this in child diets as our data are cross sectional. Collecting data at higher frequency and analysing seasonal differences in child diets could be an appropriate avenue for future research. Third, due to data limitations, we were unable to explain whether the food production is in sufficient quantity to significantly impact dietary diversity and nutritional status. However, incorporating both food quality and quantity in the analysis remains an area of our future research.

## Conclusion

As the majority of the undernourished children live in smallholder households in rural areas, understanding whether diversity in agricultural production contributes to dietary diversity and nutrition improvement especially for children is increasingly important. The present research set out to address the paucity of research to date on the relationship between agriculture production diversity and dietary diversity as well as children’s nutritional status. Our findings reveal that while positively correlated to dietary diversity score, production diversity score is negatively associated with child stunting status, but not with wasting status or zinc deficiency. This finding implies that promoting agricultural diversity should not be used as a stand-alone policy. Thus, for children’s dietary diversity and nutrition outcomes to be improved, policy makers should take a holistic approach. Promoting production diversity should be combined with other health and care programs. This study also contributes to the debate on whether market plays a larger role in enhancing dietary diversity than subsistence farming. To that end, these findings are relevant in other smallholder economies where promotion of agricultural revenues and market facilities could be an important complementary strategy to improve child dietary quality and nutritional outcomes.
